# Modification of kefir grain by yeast substitution and long-term stability of the microbiota of kefir grain

**DOI:** 10.3168/jdsc.2026-1094

**Published:** 2026-08-24

**Authors:** Hidemasa Motoshima, Ikumi Fujioka, Kenji Uchida

**Affiliations:** Research Center, Yotsuba Milk Products Co. Ltd., Kitahiroshima, Hokkaido 061-1264, Japan

## Abstract

The yeast microbiota of kefir grains is highly diverse. Whereas one type of kefir grain contains lactose-fermenting yeast, a second type may not. The presence of lactose-fermenting yeast influences the flavor and shelf life of kefir by promoting alcoholic fermentation and the attendant formation of carbon dioxide gas. To suppress gas production, we developed a method in 1994 to modify the yeast microbiota of kefir grain. The traditional kefir grain (TCHR grain) contains a lactose-fermenting yeast (*Kluyveromyces marxianus*) and 2 non-lactose-fermenting yeasts (*Saccharomyces cerevisiae* and *Kazachstania exigua*). First, we created a yeast-free NCHR grain from the TCHR grain by using an antifungal agent. The NCHR grain grew logarithmically in milk, as does the TCHR grain. The original *S. cerevisiae* yeast was then returned to the NCHR grain to create the yeast-substituted YCHR2 grain. Kefir using YCHR2 grain as a starter showed low alcoholic fermentation and suppressed package swelling, consequently improving storage stability at 10°C. Additionally, another NSB grain was obtained in 1994 that naturally contains only non-lactose-fermenting yeasts. We continued to subculture the TCHR, YCHR2, and NSB grains over 23 yr at a frequency of about once a week and analyzed the microbiota in both 1994 and 2018. Despite subtle changes in microbiota evaluation procedures between 1994 and 2018, there was no change in the fact that *Lactobacillus kefiranofaciens* and *Lentilactobacillus kefiri* constituted the dominant lactic acid bacterial microbiota in all the grains. The yeast microbiota of TCHR and YCHR2 grains were also unchanged, but that of NSB grain changed to contain lactose-fermenting yeasts, probably due to cross-contamination. The experimental results and observations show that the yeast microbiota of kefir grains is changeable and suggest that yeasts are not essential for growth of kefir grain.

## Graphical Abstract

**Figure:**
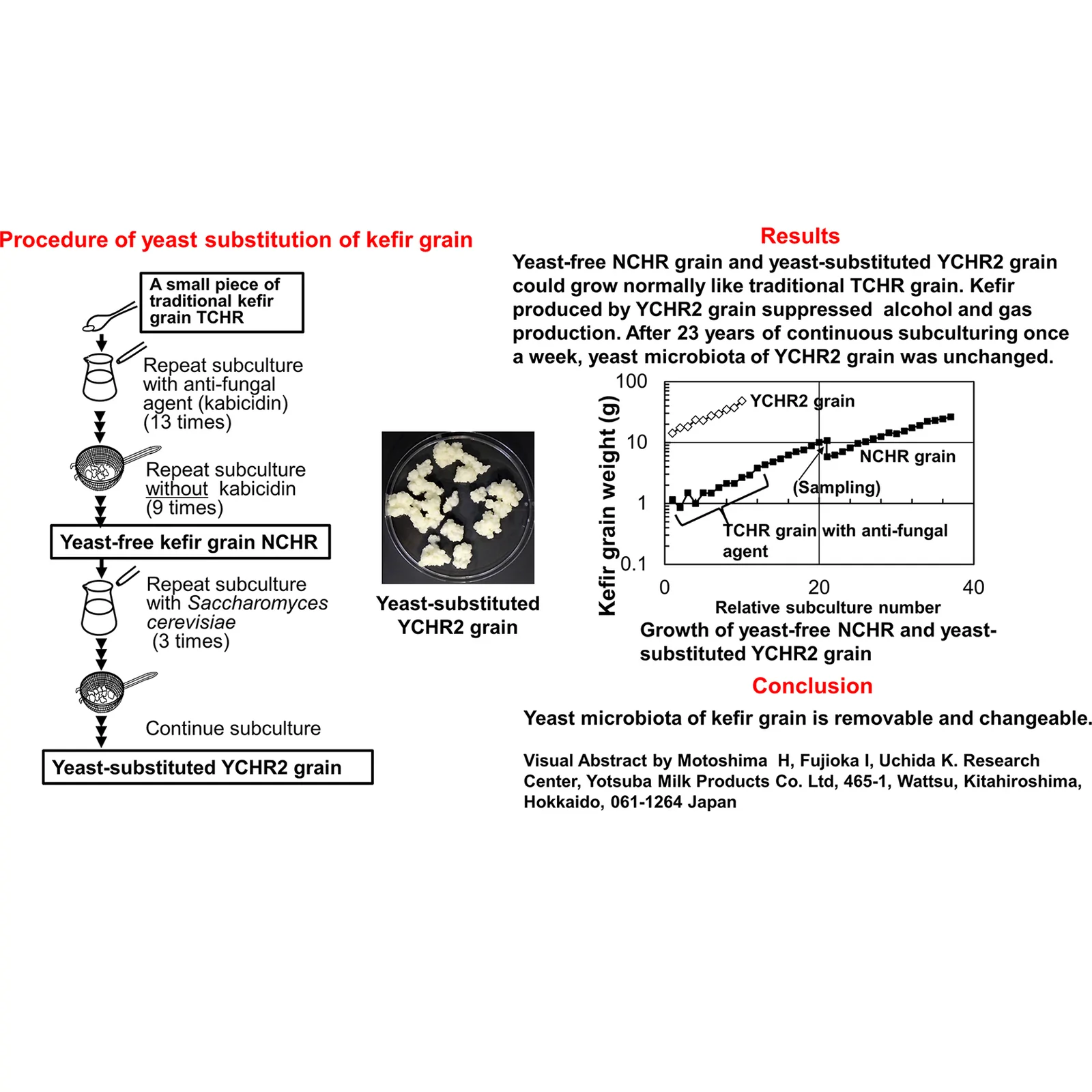

Kefir is a traditional fermented milk originating from mountain tribes inhabiting the northern side of Caucasus peaks (Kern, 1881). For the traditional manufacture of kefir, a natural starter culture known as kefir grain is used. Recently, it was revealed that kefir grains from 7 different origins mainly share a simple lactic acid bacteria (**LAB**) microbiota (Motoshima et al., 2025) consisting of *Lactobacillus kefiranofaciens* (approximately 87%) and *Lentilactobacillus kefiri* (approximately 12%) as the major components. The *Lb. kefiranofaciens* consists of 2 subspecies, *Lb. kefiranofaciens* ssp. *kefiranofaciens* (a kefiran producer, approximately 18%) and *Lb. kefiranofaciens* ssp. *kefirgranum* (a non-kefiran producer, approximately 82%). Minor LAB species are also present, including *Lentilactobacillus parakefiri*, *Leuconostoc mesenteroides*, and *Lactococcus lactis*. By contrast, the yeast microbiota of kefir grain differs strongly by origin, and in a sample of kefir grains containing both lactose-fermenting and non-lactose-fermenting yeasts, only *Kazachstania exigua* (a non-lactose-fermenting yeast) was found in every grain (Motoshima et al., 2025).

The main microbiota of kefir grain and that of kefir (the fermented milk) itself are somewhat different, with more *Lactococcus* spp. and *Leuconostoc* spp. being present in kefir (Motoshima et al., 2025). However, it is known that kefir manufactured using kefir grain still contains kefir-specific *Lb. kefiranofaciens* and *Len. kefiri* as a main component of its microbiota (Nejati et al., 2022).

According to the international food standard for kefir, kefir contains 10^7^ cfu·g^−1^ or more LAB and 10^4^ cfu·g^−1^ or more yeast. However, no essential LAB and yeast species for kefir have been defined (FAO/WHO, 2018).

During commercial production of kefir, alcoholic fermentation by the yeast present in kefir grains produces carbon dioxide in an equimolar ratio, which may cause the packaging container to swell. To produce commercial kefir with kefir grain, it is therefore necessary to suppress excessive gas formation to extend the shelf life as much as possible.

To this end, we developed a method in 1994 of substituting the yeasts of kefir grains that contain lactose-fermenting yeasts with non-lactose-fermenting yeasts, and created the yeast-substituted kefir grain YCHR2 and patented it in 2002 (Motoshima and Tsukasaki, 2002), though this grain has not yet been formally described in a scientific article. Moreover, we identified another kefir grain (NSB) in 1994 that naturally contains only non-lactose-fermenting yeasts. This NSB grain is equivalent to the YCHR2 grain created in this experiment, in that it does not contain lactose-fermenting yeast. The microbiota of TCHR, NSB grain, and yeast microbiota of YCHR2 grain filtrate were analyzed in 1994. In the ensuing years, the grains were subcultured in our laboratory about once a week, and a second analysis of the microbiota of these 3 grains was performed in 2018. Of these, the microbiota of TCHR and NSB in 2018 has already been reported (Motoshima et al., 2025).

Even if natural grains that contain only non-lactose-fermenting yeast (e.g., NSB) exist, it remains important to have a viable technique for modifying the yeast microbiota of kefir grain. This is because improvement of the yeast microbiota of kefir grain allows for improvement of the quality of the kefir produced. In this report, we detail the method of kefir grain yeast substitution conducted in 1994 and show how yeast removal affects grain growth. In addition, by comparing the microbiota of a natural kefir grain and yeast-substituted kefir grain in 1994 and 2018, we demonstrate the long-term stability of kefir grain microbiota.

The TCHR grain was purchased from Chr. Hansen A/S (Denmark) in 1981. The NSB grain was first identified in 1994 in a household in Hokkaido and was obtained for our study. The yeast-free kefir grain NCHR as well as the yeast-substituted kefir grains YCHR2 were created in this experiment.

*Saccharomyces cerevisiae* LW-1 is a non-lactose-fermenting yeast that was originally isolated from TCHR grain. Yeast was cultured in yeast malt broth in a tube at 25°C for 5 d with shaking at 150 rpm. The fresh culture was directly used as an inoculant.

Routine subculture of kefir grain was described in a previous paper (Motoshima et al., 2025). In the yeast substitution experiment, subculturing was usually performed every day except on weekends. The coagulated reconstituted skim milk (**RSM**) was filtered through a 16-mesh (mesh size approximately 1.3 mm) metal strainer, and the kefir grain collected on the strainer was used in the next subculture. The TCHR (purchased in 1981), NSB (obtained in 1994), and YCHR2 (created in 1994) used in this experiment were maintained and generally subcultured weekly until present (2026). The weight of the kefir grain increased with each subculture; therefore, some portions of the grain were occasionally removed. The grain growth rates were evaluated from the slope of the relationship of the weight to the subculture number.

In sample preparation for microbiota analysis of kefir grain used in the 1994 experiments, a piece of kefir grain was washed with sterilized saline (0.85% wt/vol NaCl) and then homogenized with 9-times weight of saline with a stomacher in a plastic bag. In the 2018 experiments, the kefir grain was washed with sterilized saline and homogenized on ice using an Ultra-Turrax T25 homogenizer (IKA Laboratechnik, Japan) at 20,000 rpm for approximately 2 min. The detailed methods have been previously reported (Motoshima et al., 2025).

In the 1994 experiments, *Lb. kefiranofaciens*, *Len. kefiri*, and *Leuconostoc* spp. were cultured in Rogosa-cheese whey (**R-CW**) agar (Kojima et al., 1993) and incubated at 30°C for 5 to 10 d under anaerobic conditions by using Mitsuoka's steel wool jar method (Benno et al., 1984). In the 2018 experiments, we used Rogosa milk whey (**RMW**) agar and Rogosa skim milk powder (**RSMP**) agar, both of which were modified from R-CW agar. The detailed methods have been previously reported (Motoshima et al., 2025). The medium for yeast in both the 1994 and 2018 experiments was **YMX** (yeast malt + X-gal) agar (Motoshima et al., 2025). For *Lactococcus* spp., M17 agar (Merck, Japan) was used, and incubated at 30°C for 5 d under anaerobic conditions.

The identification of LAB in the 1994 experiments was conducted with the standard methods available at the time (Carr and Passmore, 1979). *Lactobacillus kefiranofaciens* subspecies cannot be differentiated by 16S rDNA sequencing (Vancanneyt et al., 2004). Since the distinction between these subspecies is based on the practical phenotype of kefiran production capability, the isolates were identified to the species level using physiological methods, and the subspecies was subsequently determined based on the colony phenotype of fully developed colonies. We identified glossy and ropy colonies on R-CW as *Lb. kefiranofaciens* ssp. *kefiranofaciens* and non-glossy and ropy colonies as *Lb. kefiranofaciens* ssp. *kefirgranum*. Identification of yeasts in 1994 was performed according to physiological methods (Kreger-van Rij, 1984).

In 2018, all microorganisms including LAB and yeasts were identified with the MALDI-TOF MS method (MALDI Biotyper, Bruker, Japan). Because MALDI-TOF MS also could not distinguish the subspecies of *Lb. kefiranofaciens*, it was identified up to the species level and then the subspecies in colony form was determined as described previously (Motoshima et al., 2025).

First, the kefir grain NCHR, which contains no yeasts (yeast-free kefir grain), was created (Figure 1A). Following the methods of Shimoda et al. (1979), we prepared an antifungal kabicidin solution by dissolving 100 mg of kabicidin (Nihon Pharmaceutical Co. Ltd., Japan) in 10 mL of ethanol according to the manufacturer's protocols. Then, 0.68 mL of the solution was added to 100 mL of RSM. This mixture was placed in a conical beaker sealed with aluminum foil and pasteurized in a boiling water bath for 15 min, then cooled to room temperature. A small sample (approximately 1 g) of TCHR grain was inoculated in the RSM and incubated at 22°C until acid coagulation occurred (normally overnight), then cooled to 10°C until the next subculture. The grain was collected in a metal strainer and a further 13 rounds of subculture were performed with the addition of kabicidin as previously described. Then, 9 rounds of subculture were performed without the addition of kabicidin. Finally, after confirming the absence of yeasts in the washed grain using YMX agar, we defined the obtained sample as yeast-free NCHR grain. After 78 rounds of subculture, the absence of yeast was confirmed again using YMX agar.

**Figure 1:**
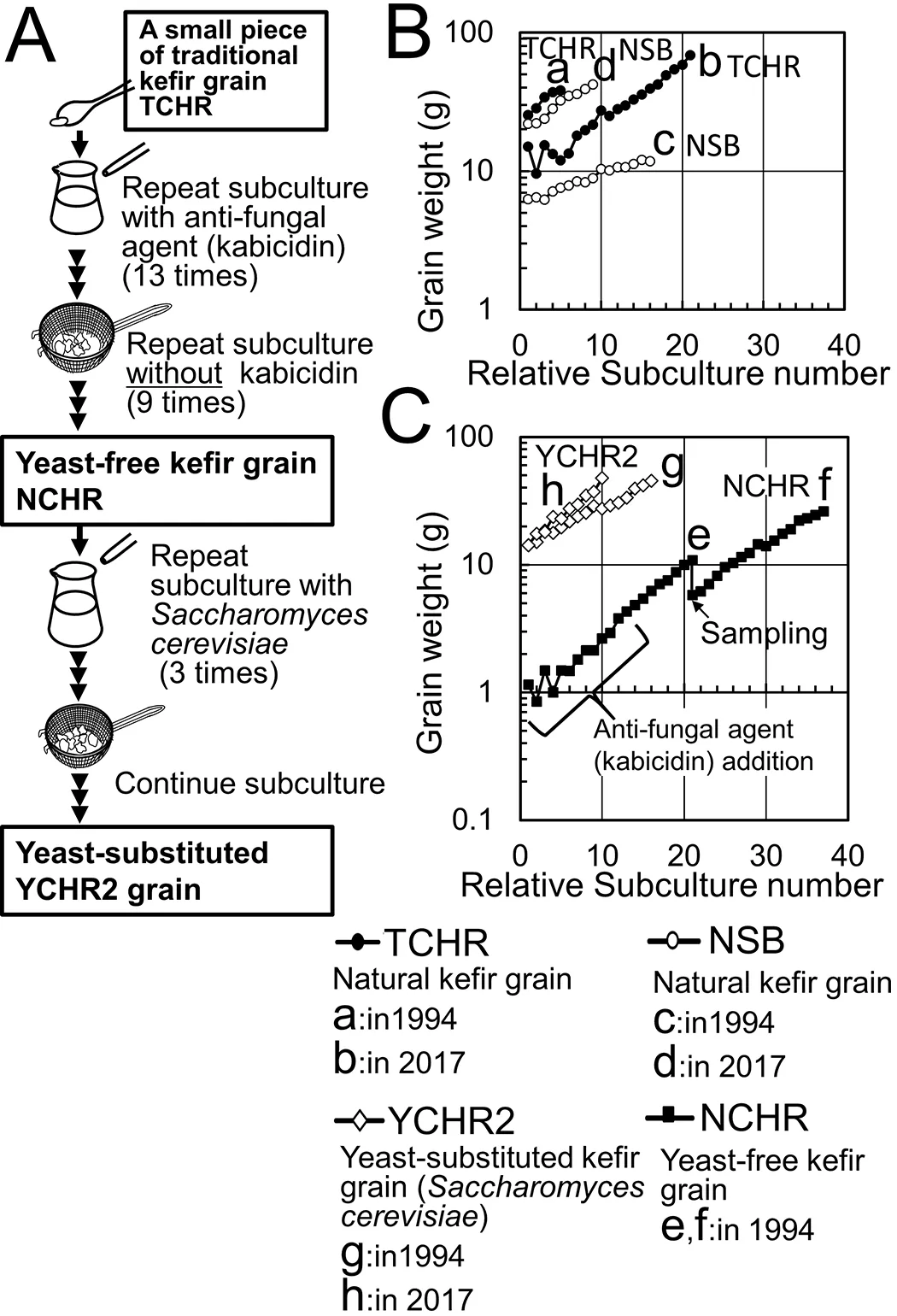
Procedure of yeast substitution experiment of kefir grain and grain growth. (A) Procedure of yeast deletion and substitution method of kefir grain. (B) Growth of traditional kefir grain TCHR and NSB. Growth line of TCHR grain in 1994 (line a), in 2017 (line b). Growth line of NSB grain in 1994 (line c) and in 2017 (line d). (C) Growth line of NCHR grain and YCHR2 grain in the yeast substitution experiment in 1994. In 1st to 13th subculture, subculture medium contains the antifungal agent kabicidin. Therefore, TCHR grain changed into NCHR grain (line e). In 21st subculture, a part of the changed yeast-free NCHR grain was used in the yeast substitution experiment, then continued to subculture (line f). Therefore, line f is that of yeast-free NCHR grain in 1994. Growth line of yeast-substituted (with *Saccharomyces cerevisiae*) YCHR2 grain in 1994 (line g) and in 2017 (line h). A detailed explanation is given in the text.

For YCHR2 grain preparation (Figure 1A), 4 mL of fresh culture of *S. cerevisiae* LW-1 grown in yeast malt broth and 14.2 g of NCHR grain were inoculated together in 400 mL of pasteurized RSM, then subcultured in a parallel manner with the routine kefir grain subculture procedure. In addition, we performed 2 subcultures with 4 mL of *S. cerevisiae* LW-1 in the same manner, for a total of 3 rounds of subculture. Then, we continued to subculture the grain without the addition of yeasts. After total 43 rounds of subculture, yeast microbiota in the filtrate obtained from the subculture was analyzed on YMX agar.

In the production of kefir for the storage stability test, the filtrate produced during subculture of TCHR grains and YCHR2 grains was used as bulk starter, respectively. Pasteurized milk (fat 2.0%, SNF 9.5%) was prepared using RSM and 47% (wt/wt) milk fat cream, homogenized at 150 kg·m^−2^ pressure using a mini-plant dairy homogenizer, pasteurized at 90°C for 10 min, and then cooled to 22°C. Each bulk starter mentioned previously was added (3 wt/wt %), then incubated overnight at 22°C. After breaking the curd, the resulting kefir was placed in a hard clear plastic package (volume 100 mL) and tightly heat-sealed with packaging film, then kept under 10°C or 15°C for the storage stability test. The reason for testing at such a high temperature is that the upper limit of the legal distribution temperature of fermented milk in Japan is 10°C. Alcohol content was measured using an enzymatic method (Bernt and Gutmann, 1974).

By modifying this TCHR grain, we created the yeast-free kefir grain NCHR. No yeasts were detected in the filtrate-obtained NCHR grain at 3 time points after the final addition of kabicidin and even in the 78th subculture; this demonstrates that NCHR was practically a yeast-free kefir grain. The yeast microbiota of the YCHR2 grain filtrate at the 43rd subculture consisted solely of *S. cerevisiae* (5.96 cfu·mL^−1^). This shows that the yeast microbiota of kefir grain can be completely removed through the use of antifungal agents to produce yeast-free kefir grain, and furthermore that it is possible to introduce another yeast species originally derived from another kefir grain to the yeast-free kefir grain.

Table 1 shows the microbiota of TCHR and NSB kefir grains in 1994 and 2018, as well as the yeast microbiota of YCHR2 grain filtrate in 1994 and microbiota of the grain in 2018. Each microbiota analysis was performed multiple times, and representative results are shown.

**Table 1:** Comparison of the major microbiota of kefir grain TCHR, NSB, and yeast-substituted kefir grain YCHR2 analyzed between 1994 and 2018^1^

Item	Grain ID and year					
	TCHR		NSB		YCHR2	
	1994	2018	1994	2018	1994	2018
Sample (grain [G] or filtrate [F])	G^2^	G^2^	G^2^	G^2^	F^3^	G^2^
*Lactobacillus kefiranofaciens* ssp. *kefiranofaciens*	7.69	8.30	8.52	7.90	—	7.60
*Lactobacillus kefiranofaciens* ssp. *kefirgranum*	7.43	9.23	7.59	8.85	—	9.09
*Lentilactobacillus kefiri*	7.20	8.60	7.26	8.40	—	8.18
*Leuconostoc* spp.	<6^4^	ND	ND	ND	—	ND
*Lactococcus lactis*	7.90	ND	7.90	6.30	—	5.30
*Kluyveromyces marxianus*	5.45^5^	6.60	ND	6.56	ND	ND
*Saccharomyces cerevisiae*	5.43	ND	ND	ND	5.96	7.94
*Kazachstania exigua*	6.81^6^	7.48	6.81^6^	5.82	ND	ND

1 Detection limits vary depending on the density of competing colonies in the same agar medium. ND: not detected; —: not estimated.

2 Log cfu∙g^−1^ (average of 2 data points).

3 Log cfu∙mL^−1^ (average of 2 data points).

4 Detected as *Leuconostoc lactis* in 1994.

5 Detected as *Candida kefyr* (synonym of *Kluyveromyces marxianus*) in 1994.

6 Detected as *Candida holmii* (synonym of *Kazachstania exigua*) in 1994.

The major composition of the LAB microbiota of TCHR grain was practically unchanged after 23 yr of subculture. The main microbiota profile of the TCHR grain included *Lb. kefiranofaciens* ssp. *kefiranofaciens*, *Lb. kefiranofaciens* ssp. *kefirgranum*, *Len. kefiri*, *K. marxianus*, and *K. exigua*. In TCHR grain, *Lc. lactis* was detected in 1994, but not in 2018. However, it was reported in TCHR grain filtrate in 2018 (Motoshima et al., 2025), suggesting that the lack of detection in our study may be attributable to low bacterial counts of *Lc. lactis* in the grain. We also detected *S. cerevisiae* in TCHR grain in 1994, but not in 2018. The variation in bacterial and yeast numbers may also be influenced by differences in sample homogenization methods (i.e., stomacher vs. ultra-disperser). Microbiota analysis of the YCHR2 grain was not performed in 1994; however, only *S. cerevisiae* was present in the yeast microbiota of the filtrate. In 2018, microbiota of YCHR2 grain was analyzed; it was confirmed that the LAB microbiota was dominated by *Lb. kefiranofaciens* and *Len. kefiri*, whereas the yeast microbiota consisted solely of *S. cerevisiae*, even after more than 23 yr of subculture. Regarding the LAB microbiota of NSB grain, *Lb. kefiranofaciens* and *Len. kefiri* were the dominant species in both 1994 and 2018, whereas changes were observed in the yeast microbiota. In 1994, only the non-lactose-fermenting *K. exigua* was detected, but in 2018, the lactose-fermenting *K. marxianus* was also detected. The reason is unclear, but it may be because the kefir grain was subcultured at the same clean bench as TCHR for more than 23 yr, and cross contamination may have occurred at some time point.

Figure 1B shows kefir grain weight plotted against the rounds of subculture of TCHR grain and those of NSB grain in 1994 and 2017. All grains exhibited logarithmic growth. Although there were significant differences in the measurement periods, grain weights, and number of measurements, there appears to be no substantial difference in the growth rates of TCHR grains between 1994 (line a) and 2017 (line b; *P* = 0.62). There appears to be no difference in the growth rate of NSB grain between 1994 (line c) and 2017 (line d; *P* = 0.12). When NCHR grain was created from TCHR grain in 1994, the growth rate of the NCHR grain was assessed (Figure 1C). During the development of the NCHR grain, the RSM medium contained the antifungal agent kabicidin from the 1st to 13th subculture (Figure 1C, line e). During these subcultures, the TCHR grain changed to NCHR. A portion of the NCHR grain at the 21st subculture was used for the yeast substitution experiment, and the growth rate of this line was also assessed (Figure 1C, line f). Yeast-free NCHR grain grew normally, and the growth rate (line f) is not changed from line e (*P* = 0.45). Growth rate of YCHR2 grain that had been introduced by *S. cerevisiae* to NCHR grain in 1994 was shown (line g). It was also not changed to that of NCHR grain (Figure 1C, line f; *P* = 0.47). Growth rate of YCHR2 in 2017 (line h) was not changed from that in 1994 (line g; *P* = 0.16). These results indicate that the yeasts in kefir grains do not significantly affect the growth rate of the kefir grains.

The results of the storage stability tests at 10°C and 15°C of kefir made with TCHR and YCHR2 grains are shown in Table 2. Severe package swelling of fermented milk like yogurt is generally caused by yeast contamination. Similarly, because kefir contains yeast, there is a risk that the yeast will produce carbon dioxide—in a molar ratio corresponding to the alcohol content—causing the package to swell or, in some cases, burst. Kefir made with TCHR grain reached an alcohol content of 0.889 wt/vol% at 10°C for 14 d, and the package also swelled strongly, whereas that of YCHR2 was 0.052% (wt/vol) with no package swelling. This experiment demonstrated that if the yeast contained in kefir consists solely of non-lactose-fermenting yeast, the generation of carbon dioxide is suppressed, thereby preventing the container from swelling.

**Table 2:** Storage stability test of kefir made by yeast-substituted kefir grain YCHR2 (with *Saccharomyces cerevisiae*) in comparison to that of traditional kefir grain TCHR^1^

Starter	Estimation (°C)	Evaluation at storage days, °C (D+0 = production day)			
		D+0	D+4	D+14	D+20
TCHR grain (control)					
pH	10	4.32	4.30	4.25	NT
	15		4.19	4.24	NT
Yeast (log cfu∙mL^−1^)	10	4.56	NT	NT	7.08
	15		NT	NT	8.00
Alcoholic content (wt/vol %)	10	0.016	0.067	0.889	NT
	15		0.092	1.657	NT
Total lacticacid bacteria (log cfu∙mL^−1^)	10	8.32	NT	7.43	7.56
	15		NT	7.30	7.08
Package swelling	10	No	No	Swell	Swell
	15		Swell	Swell	Swell
Yeast substituted YCHR2 grain (with *Saccharomyces cerevisiae*)					
pH	10	4.31	4.22	4.32	NT
	15		4.20	4.34	NT
Yeast (log cfu∙mL^−1^)	10	6.14	NT	NT	6.53
	15		NT	NT	6.46
Alcoholic content (wt/vol %)	10	0.047	0.055	0.052	NT
	15		0.073	0.082	NT
Total lactic acid bacteria^2^ (log cfu∙mL^−1^)	10	8.48	NT	7.00	7.64
	15		NT	7.30	7.04
Package swelling	10	No	No	No	No
	15		No	No	No

1 NT = not tested.

2 Total lactic acid bacteria were counted using BCP plate count agar according to the official method of the Japanese ministerial ordinance for fermented milk (Ministry of Health, Labor, and Welfare, Japan, 1951; details not shown).

However, the natural NSB kefir grain obtained in 1994 also contains only non-lactose-fermenting yeast, and alcoholic fermentation seems to be suppressed. In another experiment conducted in 1994, the alcohol contents of kefir produced using TCHR and NSB grains were 0.016% and 0.025% (wt/vol), respectively, on the first day after manufacture, and 0.869% and 0.044% (wt/vol) after 14 d at 10°C, respectively. It appears that natural kefir grain without lactose-fermenting yeast has the same properties as the yeast-substituted kefir grain created during this experiment. However, the current NSB grain contains lactose-fermenting *K. marxianus*, and it does not appear to differ from kefir produced with the TCHR grain. When kefir was produced using yeast-free NCHR grain, alcohol and gas production did not occur; it was judged to have lost the refreshing flavor by experienced evaluators (however, this was not based on evaluations by multiple panelists). In contrast, kefir produced using YCHR2 grains exhibited a refreshing flavor comparable to that of kefir made with natural NSB grains, which do not contain lactose-fermenting yeast. Consequently, yeast was considered an essential component, as it exerts a profound influence on the flavor characteristics of kefir, particularly its refreshing quality. Furthermore, because substituting the yeast in this manner does not affect the dominant LAB microbiota, it is considered that the functionality of the kefir produced using it remains unchanged. Many commercial kefir products are manufactured using commercial kefir starters that do not contain yeasts. For example, commercial kefir products sold in the United States are presumably manufactured with such formulated starters; that is, *Streptococcus thermophilus* is the main microbial component (Metras et al., 2021) and neither *Lb. kefiranofaciens* nor *Len. kefiri* are detected.

The essential microbiota of kefir has not been explicitly defined; therefore, fermented milk that does not contain kefir-specific LAB can be sold under the brand name “kefir.” Considering that many consumers buy kefir in anticipation of its specific health benefits, these products may be deceptive. Therefore, to include these kefir-specific LAB such as *Lb. kefiranofaciens* and *Len. kefiri* in the final product, it is practical to use yeast-substituted kefir grain as a starter. However, further research is also required to determine whether such yeast substitution affects the nutraceutical functionality of kefir.

In this experiment, we depended on a method for the elimination of all yeasts from kefir grain described by Shimoda et al. (1979), who reported that this method had little effect on the growth of Delft kefir grain (Shimoda et al., 1979). Recently, Chen et al. (2021) have also reported that the elimination of yeasts from Tibetan kefir grain had no effect on the structure and growth characteristics of the grain. It is generally believed that kefir grain requires the symbiosis of LAB and yeast, but these reports, together with our experimental results, call this belief into question.

In the work reported here, the kefir grains were maintained under aseptic conditions on a clean bench. Therefore, the yeast microbiota of kefir grain could be maintained over the long term as long as there was no cross-contamination. However, kefir grain is ordinarily subcultured under nonaseptic conditions in the home, so contamination with yeast can easily occur. Therefore, the yeasts in kefir grain could have originated as contaminants, as is the case in yogurt. This is supported by the high diversity of the yeast microbiota in kefir grains of different origins.

In conclusion, the present study shows that the yeasts in kefir grain can easily be removed and substituted. The absence of yeast microbiota in kefir grain did not affect the growth rate or morphology of the kefir grain. By introducing only non-lactose-fermenting yeast into kefir grain, it was possible to suppress package swelling while maintaining the refreshing flavor of kefir. It was shown that the LAB microbiota of kefir grain is extremely stable after long-term subculture, and *Lb. kefiranofaciens* and *Len. kefiri* were the main microbiota. However, the yeast microbiota can be changed.

## References

[bib1] Benno Y., Sawada K., Mitsuoka T. (1984). The intestinal microflora of infants: Composition of fecal flora in breast-fed and bottle-fed infants. Microbiol. Immunol..

[bib2] Bernt E., Gutmann I., Bergmeyer H.A. (1974). Methods of Enzymatic Analysis.

[bib3] Carr J.G., Passmore S.M., Skinner F., Lovelock D. (1979). Society for Applied Bacteriology.

[bib4] Chen Z., Liu T., Ye T., Yang X., Xue Y., Shen Y., Zhang Q., Zheng X. (2021). Effect of lactic acid bacteria and yeasts on the structure and fermentation properties of Tibetan kefir grains. Int. Dairy J..

[bib5] FAO/WHO (2018).

[bib6] Kern E. (1881). Ein neues milchferment aus dem Kaukasus. Bull. Soc. Nat. Moscow.

[bib7] Kojima S., Takizawa S., Tamura S., Fujinaga S., Benno Y., Nakase T. (1993). An improved medium for the isolation of lactobacilli from kefir grains. Biosci. Biotechnol. Biochem..

[bib8] Kreger-van Rij N.J.W. (1984).

[bib9] Metras B.N., Holle M.J., Parker V.J., Miller M.J., Swanson K.S. (2021). Commercial kefir products assessed for label accuracy of microbial composition and density. JDS Commun..

[bib10] Ministry of Health, Labor, and Welfare, Japan (1951).

[bib11] Motoshima H., Fujioka I., Uchida K. (2025). Identification of dominant species common to kefir grains from seven origins for kefir grain reconstruction. J. Dairy Sci..

[bib12] Motoshima, H., F. Tsukasaki. 2002. Method for preparing kefir grain and production of fermented dairy product using the same. Japanese Pat. No. JP2002253115A.

[bib13] Nejati F., Capitain C.C., Krause J.L., Kang G.-U., Riedel R., Chang H.-D., Kurreck J., Junne S., Weller P., Neubauer P. (2022). Traditional grain-based vs. commercial milk kefirs, how different are they?. Appl. Sci. (Basel).

[bib14] Shimoda T., Arai N., Ohi A. (1979). Study on the fermented milk. J. Sagami Women's Univ..

[bib15] Vancanneyt M., Mengaud J., Cleenwerck I., Vanhonacker K., Hoste B., Dawyndt P., Degivry M.C., Ringuet D., Janssens D., Swings J. (2004). Reclassification of *Lactobacillus kefirgranum* Takizawa *et al*. 1994 as *Lactobacillus kefiranofaciens* subsp. *kefirgranum* subsp. nov. and emended description of *L. kefiranofaciens* Fujisawa *et al*. 1988. Int. J. Syst. Evol. Microbiol..

